# Dual-Task Exercise to Improve Cognition and Functional Capacity of Healthy Older Adults

**DOI:** 10.3389/fnagi.2021.589299

**Published:** 2021-02-16

**Authors:** Naina Yuki Vieira Jardim, Natáli Valim Oliver Bento-Torres, Victor Oliveira Costa, Josilayne Patricia Ramos Carvalho, Helen Tatiane Santos Pontes, Alessandra Mendonça Tomás, Marcia Consentino Kronka Sosthenes, Kirk I. Erickson, João Bento-Torres, Cristovam Wanderley Picanço Diniz

**Affiliations:** ^1^Neurodegeneration and Infection Research Laboratory, Institute of Biological Science/João de Barros Barreto University Hospital, Federal University of Pará, Belém, Brazil; ^2^Physical Therapy and Occupational Therapy Program, Federal University of Pará, Belém, Brazil; ^3^Department of Psychology, University of Pittsburgh, Pittsburgh, PA, United States

**Keywords:** cognitive dysfunction, rehabilitation, physical therapy modalities, aged, neuropsychological tests, healthy aging, dual-task exercise, physical fitness

## Abstract

**Background:**

It has been suggested that physical inactivity and lack of stimulating cognitive activity are the two most significant modifiable risk factors to impair cognitive function. Although many studies that investigated the cognitive effects of physical exercise and cognitive stimuli in dual-task conditions showed improved cognitive performance, others have not confirmed these findings. The main aim of the present work is to analyze the effects of a dual-task multimodal physical exercise training, at moderate intensity, and cognitive stimulation on cognitive and physical function in healthy older adults.

**Methods:**

This clinical trial was registered on the Brazilian Registry of Clinical Trials (RBR-9zrx3d). Here we tested the effects of a dual-task multimodal physical exercise training, at moderate intensity, on cognitive and physical function and quality of life in community dwelling older adults. The training protocol included 24 group sessions, 2/week, per 75 min. Cognition was assessed using CANTAB automated neuropsychological tests and Functional Capacity to Exercise tests. Performance was compared from baseline to post intervention and to a non-exercise control group using Mixed Linear Model for repeated measures.

**Results:**

Control (CG) and dual-task (DTEx) groups progressed differentially over time on performance of episodic memory, sustained visual attention, functional mobility, cardiorespiratory fitness, lower limbs strength resistance, agility, quality of life and dual-task performance with significant improved DTEx performance. Control group did not show any significant changes on these tests except for showing a reduction in dual-task performance.

**Conclusion:**

We suggest that the dual-task combination of multisensory cognitive stimulation and multimodal moderate physical exercise training, twice a week, may be adopted as an effective program to reduce progression of age-related cognitive decline and improve physical fitness and quality of life on healthy older adults.

**Clinical Trial Registration:**

Brazilian Registry of Clinical Trials: https://ensaiosclinicos.gov.br/rg/RBR-9zrx3d -UTN code: U1111-1233-6349.

## Introduction

As we celebrate greater longevity, we are witnessing a change in the nosological profile that now includes higher incidence rates of neurodegenerative diseases associated with aging, including cognitive decline and dementia, of which the prevalence doubles every 6 years from the age of 65 (Cheng, 2016). Dementia implies high costs for health systems and great negative impact on quality of life for patients, as well as family members and caregivers (Prince et al., 2015). Further aggravating this scenario is that during the course of the last few generations there is greater availability of information technologies and mobile telecommunications with less expensive internet access, which has expanded the underlying trend of sedentary behavior (Owen et al., 2020). The increasing time spent sitting or lying down, sitting in automobiles or public transportation, combined with increased life expectancy, contributes to the increased risk and prevalence of type 2 diabetes (Garneau and Aguer, 2019), cardiovascular disease (Morris et al., 1953; Green et al., 2017), cancer (Ninot et al., 2020), musculoskeletal disability (Rezuş et al., 2020), as well as a broad range of other adverse health outcomes related to a sedentary lifestyle behavior (Owen et al., 2020) including the long term risk of dementia (Magnon et al., 2018; Ekblom et al., 2019; Coelho et al., 2020) and other age-associated neurodegenerative diseases (Kivimäki et al., 2019).

It is estimated that by 2050 the number of people living with dementia worldwide will increase from 47 to 132 million (Prince et al., 2015; Irwin et al., 2018). In line with this prediction, the global number of people living with dementia more than doubled from 1990 to 2016, mainly due to increases in population aging and growth (Nichols et al., 2019). Thus, healthy aging to prevent neurodegenerative diseases has become increasingly important for public health policy makers, and until breakthroughs are made in prevention or curative treatments, dementia will constitute an increasing challenge to health care systems worldwide (Nichols et al., 2019). Given the multifactorial etiology of dementia, simultaneous interventions in multiple domains might be critical for minimizing effects of various risk factors (Kivipelto et al., 2018) and for prevention and treatment of age-related cognitive decline.

Exercise programs are viable interventions to improve cognition and reduce cognitive decline (Stillman et al., 2020). It is an efficient, safe, and low-cost non-pharmacological strategy broadly investigated: aerobic and strength training or the combination of both, show positive effects on brain structure and function, behavior, and cognition (Erickson et al., 2019). Recommendations suggest multimodal training (Chodzko-Zajko et al., 2009; Gregory et al., 2013; Northey et al., 2018; Wollesen et al., 2020). This recommendation is based on the different types of training contributions to cognition (Liu-Ambrose and Donaldson, 2009; Forte et al., 2013) and physical function (Cadore and Izquierdo, 2013). It is suggested that physical training should be heart rate monitored and performed at moderate intensity (Lauenroth et al., 2016; Stojan and Voelcker-Rehage, 2019).

The differential efficiency of dual-task intervention in comparison to single stimulation is unclear (Bruderer-Hofstetter et al., 2018; Guo et al., 2020), but in healthy older adults the simultaneous conduction of a mental and a physical task (dual-task) seems to be particularly efficient to increase cognitive function (Eggenberger et al., 2015; Falbo et al., 2016; Lauenroth et al., 2016; Tait et al., 2017; Gheysen et al., 2018; Herold et al., 2018), in particular if mentally challenging activities are simultaneously performed with multimodal physical exercises (Gheysen et al., 2018; Li et al., 2018; Northey et al., 2018). However, because of the wide variation between protocols, the lack of detailed methodological description (e.g., baseline levels of physical fitness, and training intensity) and small sample sizes in some studies (Stojan and Voelcker-Rehage, 2019; Guo et al., 2020), there is still a need for more research for an optimal protocol and information on detraining effects (Gheysen et al., 2018).

Accurate and precise tools for assessing cognition contribute to more robust inferences about the influence of interventions on cognition. In addition, it is necessary to assess cognitive functions that are ltered across lifespan, such as episodic memory (Korkki et al., 2020), working memory (Cansino et al., 2020) and attention (McDonough et al., 2019). To go for it, we used automated computerized neuropsychological tests with great specificity and sensitivity (De Jager et al., 2002; Wild et al., 2008; Cabral Soares et al., 2015). Compared with traditional standard methods, automated computer tests minimize floor and ceiling effects, standardize the format of application, and measure the speed and accuracy of responses with greater sensitivity and specificity (Wild et al., 2008).

Because cognitive effects of exercise and cognitive stimulation in dual-task paradigms are ambiguous (Ansai et al., 2017; Tait et al., 2017; Guo et al., 2020; Wollesen et al., 2020) there is a need for more sensitive cognitive testing batteries rather than using screening tests as outcome measures to assess the influence of dual-task interventions (Wollesen et al., 2020). Thus, with a highly sensitive evaluation battery, we have gathered quantitative and qualitative characteristics based on the most recent recommendations to investigate whether a dual-task protocol following these guidelines is effective. We hypothesized that a moderate-intensity multimodal exercise program performed simultaneously to a complex previously validated cognitive stimulation is an effective intervention program to improve verbal and visual episodic memory, sustained visual attention, physical function, and quality of life of healthy older adults. We expected to find that control (CG) and dual-task (DTEx) groups would progress differentially over time on performance of physical and cognitive tasks.

## Materials and Methods

### Overview

This clinical trial was registered on the Brazilian Registry of Clinical Trials (RBR-9zrx3d). Participants were allocated not randomly to either the Dual-Task Exercise (DTEx) or Control (CG) groups. All participants were given the opportunity to choose to take part into DTEx or CG prior to baseline assessments, based on their own possibility to come to the intervention facility and stay committed with regular session attendance. The factors that determined the participants choice on groups included the home distance to intervention facility (the main reason in the most cases), incompatibility with the training session schedule and, for a small number of participants also reported difficulties in transportation to intervention site. The DTEx participated in a 24-session intervention protocol with physical exercise training and simultaneous cognitive stimulation (Dual-task). The Control (CG) group participants received educational materials on health-related topics. Both groups were instructed to maintain their daily routine. Both groups were evaluated before and after a 3-month intervention period. This study was approved by the Health Science Institute of Federal University of Pará Review Board (CAAE no. 03427318.3.0000.0018).

### Participants

Participants were community dwelling, healthy older adults invited to participate by advertisements on social media, seniors’ centers, health care units and the University surrounding community. To be eligible, participants were required to be >59 years old and cognitively healthy according to a Mini Mental State Examination (MMSE; 0–30 points) cutoff score adjusted to educational levels for the Brazilian population as follows: illiterate, 13; 1–7 years of schooling, 18; ≥8 years of schooling, 26 (Bertolucci et al., 1994). They also had to report no history of traumatic brain injury, stroke, or depression and be physically inactive (no regular practice of physical exercise) for at least 6 months prior to the assessments. In addition, visual acuity of 20/30 or better (Snellen test) was adopted as inclusion criteria to reduce possible biases in cognitive performance associated with visual impairments. All participants provided written informed consent before data collection. We also required written statements from the primary care physician indicating that the participant was safe to participate in an exercise program.

To determine the appropriate sample size needed to test our aims, we used G Power 3.1 software^1^ using *a priori* power analysis. Considering that previous meta-analyses have reported small-to-moderate sized effects of physical exercise on cognitive performance in older adults (Colcombe and Kramer, 2003; Zhu et al., 2016), we estimated a conservative effect size of *f* = 0.20. We based the calculation on a statistical power of 85%, a two-sided hypothesis test, an alpha level of 0.05 and an analysis of variance model, within-between interaction. Sample size calculation resulted in at least 60 participants, 30 in each group. Estimating 20% dropout, the final size was calculated at 36 participants per group.

**FIGURE 1:**
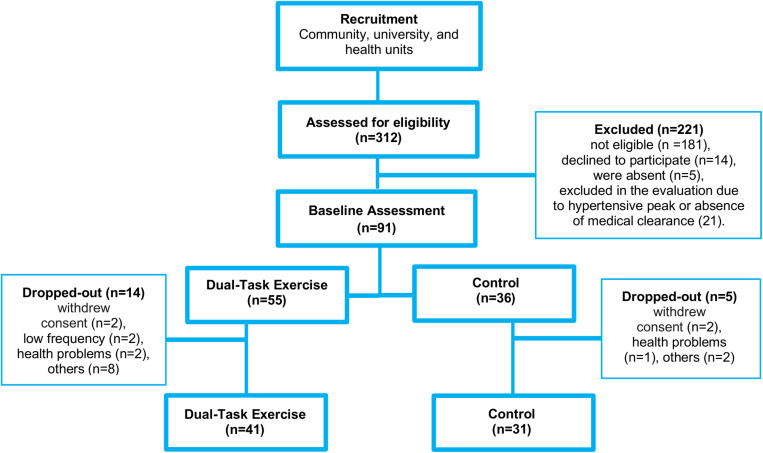
Flow diagram of recruitment, allocation, intervention, and final sample included in data analysis.

Participants completed all the cognitive, functional exercise capacity tests and quality of life assessments in a single day, before and after the intervention period. On average, tests were completed within 90 min and a brief break was offered between tests. Initially, 312 older adults were invited to participate, of which 221 were excluded based on eligibility criteria, not consenting to the procedures, or absence to the scheduled assessment. Thus, 91 participants were divided into DTEx (*n* = 55) and CG (*n* = 36). Over the 3-month intervention period, 14 participants from the intervention group and 5 from the CG dropped-out to participating in fewer than 75% of the sessions, health problems unrelated to the intervention, withdrawal from the study, or other reasons. The final analysis included 41 older adults in DTEx and 31 in CG (Figure 1). Data were collected at the Federal University of Pará Facilities, in the same testing room with control for noise, luminosity and temperature.

### Cognitive Assessment

The MMSE was used for eligibility purposes as described above (Bertolucci et al., 1994, 2001). The CERAD Word List Memory was used to assess episodic verbal memory (immediate memory and word list delayed recall) and recognition memory. The tests were applied according to Bertolucci et al. (1998).

Cambridge Neuropsychological Test Automated Battery (CANTAB) is an automated test battery used for cognitive assessment in the present study. CANTAB is less influenced by the administrator due its non-verbal stimuli and responses required. In addition, they are visually attractive and have an immediate feedback tool similar to games, which maintains motivation. It is also possible to estimate response times with millisecond precision, avoiding the compression of scales (Sahakian and Owen, 1992). In addition, studies suggest that automated tests are a more sensitive tool for identification of subtle variations in cognitive performance (De Jager et al., 2002; Wild et al., 2008). The Motor Screening Test (MOT) was performed to assess the ability to understand and complete tasks using the touchscreen technology.

Episodic memory was assessed by PAL. During the PAL test, 8 boxes were uncovered, one by one, in a randomized order, to reveal either an empty space or a figure. Then each figure was individually displayed on the center of the screen and the participant was instructed to indicate the location in which the figure was originally exposed. In case of error, the figures were presented again for up to 10 attempts. Initially, 2 figures were presented, and, to the extent of success, the number of figures increased to 3, 6 and then up to 8. PAL test administration time is around 7–10 min, but largely depends on the participant’s performance and number of repeat presentations required. There is no limited response time and because of that no predetermined inter-stimulus interval. PAL output variables included: PAL Stages completed (PAL SC), which corresponds to the number of completed steps, and is a key result of test success, a higher score is better; PAL Number of patterns succeeded (PAL NPS), which corresponds to the number of pairs successfully completed in the last stage that the participant performed, a higher score is better; and PAL Total Trials Adjusted (PAL TTA), which represents the total number of figure presentations required for the participant to answer correctly (maximum 10 presentations), a lower score is better (Cambridge Cognition, 2016).

RVP assesses the ability to sustain visual attention. In a central rectangle, several subsequent and random numbers were displayed, at the rate of 100 digits per minute. In the training stage, the volunteer was instructed to press a pad after a target number sequence (3–5–7). In the test stage, the participant was instructed to press the pad after any of three target sequences (3–5–7, 2–4–6, or 4–6–8). The administration time is around 7 to 10 min. Output measurements were RVP Mean latency, which details the average response time in milliseconds. This measure only includes correct responses within an 1,800 ms response window. RVP latency is a good indicator of sustained attention (Cognition, 2014).

### Physical Activity Assessment

Self-reported physical activity data were collected using the International Physical Activity Questionnaire (IPAQ, version 8, long form) in an interview (Benedetti et al., 2004). The IPAQ consists of 27 questions that quantifies physical activity, in a typical week, considering only activities performed for at least 10 min. Participants were asked to report the time spent in physical activity performed across work, domestic activities, exercise/leisure time, and transport at each of 3 intensities: walking, moderate, and vigorous. Scoring procedures followed the Guideline for data processing and Analysis of the IPAQ^2^. Results are expressed in Metabolic Equivalents-minutes per week (MET min/week).

### Functional Exercise Capacity Assessment

Functional exercise capacity assessments included measures of Functional Mobility (Timed Up and Go Test–TUG), an indirect measure of cardiorespiratory fitness (Six-Minute Walk Test–6MWT), lower limb strength resistance (30-seconds Chair Stand Test–30 CST). Briefly: TUG measures the total time (in seconds) the participant rises from an armless chair, walks 3 m, and returns to sit position (Podsiadlo and Richardson, 1991); 6MWT assesses the distance walked on a flat, hard surface, in a period of 6 min. The participants were instructed to walk at their own pace. Speed changes were allowed. Standardized incentive phrases were used throughout the test. The total distance walked (in meters) during 6 min was used for performance comparison between groups (American Thoracic Society, 2002). On the 30 CST test, the number of times the participant gets up from a sitting position on an armless chair is recorded for 30 s. If necessary, ergonomic adjustments were made to ensure proper positioning (Jones et al., 1999).

Walking while talking test (Hall et al., 2011) was used to assess dual-task Agility by using walking speed during the task and verbal fluency, as a cognitive addition to the motor task. Agility was also measured by walking speed as a single task.

### Quality of Life Assessment

Quality of life was assessed using the 36-Item Short Form Health Survey (SF-36). It is a self-reporting multidimensional instrument for routine monitoring and assessment of care outcomes in adults, encompassed in 8 scales or domains: functional capacity, physical aspects, pain, general health status, vitality, social aspects, emotional aspects and mental health, each domain assumes values from 0 (worst) to 100 (best) (Ciconelli et al., 1999).

### Physical Exercise in a Dual-Task Intervention Program

The intervention program was composed of 24 intervention group sessions. Each group had a maximum of 15 participants and took place at community centers. Training sessions included warm-up (10 min), aerobic exercise (30 min), resistance exercise (30 min), and stretching (5 min). In all sessions, participants used individual cardiofrequencimeter (Polar FT1 Heart Rate Sensor) to monitor the cardiovascular training zone and were encouraged to exercise at a moderate intensity (60–70% of the maximum heart rate estimated using the Karvonen formula).

The warm up was performed by walking at different speeds associated with upper limbs movements. Aerobic training was performed in alternating sessions, consisting of walking exercises associated with a functional circuit, agility (zigzag between cones, carrying weights from one end to the other) and gait. Balance (walking on different surfaces, single leg support) and coordination exercises (throwing a ball into a basket, walking while passing a ball under the legs) were also performed. In the alternative session the participants practiced a dance choreography that would be resumed the following week. Regional musical styles that were pleasing to the participants were used. The cognitive training component, aerobic, balance and coordination tasks of the Dance training was met with the choreography learning and execution itself.

Resistance training prioritized multiarticular and global exercises such as squat and bench press. Periodization was defined according to American College of Sports Medicine recommendations, increasing the number of repetitions every 2 weeks starting with 3 series of 10 repetitions followed by 3 series of 15 repetitions and increased workload on 3 series of 10 repetitions. On session 12 changes to the training program were done and periodization progress repeated until session 24 (Garber et al., 2011). It is important to note that all exercise sessions were performed simultaneously with cognitive tasks. The full description of the Dual-Task intervention Program is described in Table 1. All sessions were run by two no blinded researchers with degrees as Physical Therapist and Bachelor’s in Physical Education. Two Health/Sport Science graduate students helped during the sessions.

**TABLE 1:** Dual-task intervention program protocol.

Sessions	Stimulus	Cognitive tasks simultaneous to exercise	Exercise
1	Speech	The group was encouraged to speak out loudly the days of the week, months of the year, and the alphabet in direct and reverse order.	Functional Circuit + Walking
	Long-term memory	Participants were asked to remember pre-selected words and sing songs with the vocabulary included.	Resistance training
2	Short-term memory	At functional physical exercise circuit beginning, researcher reads a word sequence (places, animals, and objects), which were requested to be reproduced by the participant at the circuit endpoint.	Functional Circuit + Walking
	Mathematical thinking	Simple calculations (arithmetic).	Resistance training
3	Motor learning	Learning a new choreography	Dance
	Semantic and phonological fluency	The group was encouraged to remember and to speak out loud words from a certain category or phoneme.	Resistance training
4	Mathematical thinking	Simple calculations (arithmetic).	Functional Circuit + Walking
	Reasoning	The participants were encouraged to deduce a hidden word on the board. Researchers provided clues.	Resistance training
5	Long-term memory and Motor learning	Remember the previous choreography	Dance
	Speech	Participants were asked to do storytelling and, collaboratively, create a new story.	Resistance training
6	Attention, Decision-making and Short-term memory	Specific sound stimulus (one whistle, two whistles, and applause) were associated with a specific sequence of motor tasks to be performed.	Functional Circuit + Walking
	Short-term memory and Attention	One of the participants started a sequence of grocery store shopping list saying: “I went to the grocery and bought an…” (e.g., apple). The closest participant was asked to repeat the previous statement and add a new item to the shopping list. The process was continuous and items cumulative until everyone in the group had contributed.	Resistance training
7	Motor learning	Learning a new choreography	Dance
	Reasoning	The participants were encouraged to deduce a hidden word on the board. Randomly selected participant provided clues.	Resistance training
8	Attention, decision-making, and inhibition	At the sound of a whistle, participants should perform a sequence of pre-learned motor tasks. At the sound of two whistles, they should perform the movement illustrated among other equal distractors from a projected image.	Functional Circuit + Walking
	Inhibition and Processing speed	Stroop Test	Resistance training
9	Long-term memory and motor learning	Remember the previous choreography	Dance
	Long-term memory and autobiographical	Old singers’ photos and songs were placed, and the group was asked to identify the singer, named the song, and asked to share personal experiences evoked by the music.	Resistance training
10	Attention, decision-making, and short-term memory	Specific sound stimulus (one whistle, two whistles, and applause) were associated with a specific sequence of motor tasks to be performed.	Functional Circuit + Walking
	Speech	The group was encouraged to speak out loudly the days of the week, months of the year and the alphabet in direct and reverse order.	Resistance training
11	Motor learning	Learning a new choreography	Dance
	Mathematical reasoning	Simple mathematical problems	Resistance training
12	Short-term memory	At functional physical exercise circuit beginning, researcher reads a word sequence (places, animals, and objects), which were requested to be reproduced by the participant at the circuit endpoint.	Functional Circuit + Walking
	Speech	Participants were asked to do storytelling and, collaboratively, create a new story.	Resistance
13	Long-term memory and motor learning	Remember the previous choreography	Dance
	Inhibition and processing speed	Stroop Test	Resistance training
14	Long-term memory and short-term memory	At functional physical exercise circuit beginning, specific sequence of olfactory stimuli were presented. Later, the identification and the sequence of the odors presented were requested.	Functional Circuit + Walking
	Short-term memory and attention	One of the participants started a sequence of grocery store shopping list saying: “I went to the grocery and bought an …” (e.g., apple). The closest participant was asked to repeat the previous statement and add a new item to the shopping list. The process was continuous and items cumulative until everyone in the group had contributed.	Resistance training
15	Motor learning	Learning a new choreography	Dance
	Attention and short-term memory	The researcher reads news and proceeds a group discussion about the information read.	Resistance training
16	Attention and long-term memory	A song was played during the exercise, and then suddenly interrupted. Participants were encouraged to continue to sing, completing the song.	Functional Circuit + Walking
	Inhibition and processing speed	Stroop Test	Resistance training
17	Long-term memory and motor learning	Remember the previous choreography	Dance
	Attention and short-term memory	A music was played and after that the group was asked to identify among projected images, the objects mentioned by the music. Confounders images were used.	Resistance training
18	Long-term memory and short-term memory	Commemorative dates were said at the beginning of the functional circuit and requested to be mentioned at the end.	Functional Circuit + Walking
	Emotional prosody	Identification of emotions on the images displayed.	Resistance
19	Motor learning	Learning a new choreography	Dance
	Attention and reasoning	One word was given for a random participant, who had to describe characteristics of his word so the others could try to guess it.	Resistance training
20	Long-term memory and short-term memory	At functional physical exercise circuit beginning, Specific sequence of olfactory stimuli were presented. Later, the identification and the sequence of the odors presented were requested.	Functional Circuit + Walking
	Short-term memory	Visual memory game with increased degree of difficulty.	Resistance
21	Long-term memory and motor learning	Remember the previous choreography	Dance
	Long-term memory and autobiographical	Old singers’ photos and songs were placed, and the group was asked to identify the singer, named the song, and asked to share personal experiences evoked by the music.	Resistance training
22	Sustained attention and decision-making	At a specific sound, the group should change the motor task. There were confounding sounds.	Functional Circuit + Walking
	Long-term memory and reasoning	Questions about general knowledge issues.	Resistance training
23	Motor learning	Learning a new choreography	Dance
	Emotional prosody	Identification of emotions on the images displayed.	Resistance
24	Long-term memory and Motor learning	Remember the previous choreography	Dance
	Semantic and phonological fluency	The group was encouraged to remember and to speak out loud words from a certain category or phoneme.	Resistance training

### Cognitive Stimulation

The cognitive stimulation protocol associated with physical exercise included a multisensorial stimuli protocol based on a previous study published by our group (De Oliveira et al., 2014) (Table 1). To generate a multisensory stimulation, a complex program was developed including visual, auditory, and olfactory stimuli activities. The tasks mainly included functional responses to sensory stimuli, verbal and visual memory, motor learning, speech, attention, inhibition, and semantic and phonological fluency and were performed simultaneously to physical exercise training.

### Statistical Analysis

The primary aim of this study was to investigate improvements in cognitive performance for the DTEx group in comparison to the CG. Secondary outcomes were functional exercise capacity and quality of life measures.

Prior to statistical analysis, outlier values (±2 SD from the mean) were excluded. Age, education, and physical activity level (IPAQ) were compared using the Student *t*-test or Mann–Whitney based on the distribution. Differences between groups in relation to changes in cognitive, physical and quality of life parameters before and after the intervention were carried out using a Mixed Linear Model for repeated measures, in which time was considered as a within-subjects factor (pre and post) and group was treated as a between subjects factor (DTEx, CG). Bonferroni was used as a *post hoc* test. Effect size, as a measure of Intervention effect, were estimated. For this, the individual final pos-test score was subtracted from the group pre-test score average and divided by the standard deviation of the pre-test score (Sink et al., 2015). Thus, a *Z* score was obtained for each outcome measure for each group. To compare DTEx and CG interventions effects (*z* score) an analysis of covariance ANCOVA was performed adjusting for age, education, and sex, using only the group (DTEx, CG) as the factor of analysis. For outcome measures (PAL TTA, RVP ML, and TUG) in which a negative effect was indicative of better test performance any negative effect sizes were multiplied by (−1) to facilitate interpretation. Effect sizes were classified based on *Z* Score: 0.00–0.19–trivial; 0.20–0.49–small, 0.50–0.79–moderate; ≥0.80–large (Cohen, 1992). Analyses were performed using IBM SPSS Statistics version 20 (Armonk, Nova York: IBM Corporation).

## Results

Seventy-two participants were included in the final analysis: 41 (36 female) in the DTEx and 31 (25 female) in the CG. Groups were matched for age (DTEx = 67.39 ± 0.90, CG = 67.87 ± 0.99, *p* = 0.56 years), education (DTEx = 8.64 ± 0.63, CG = 8.64 ± 0.79, *p* = 0.99 years) and physical activity level (DTEx = 626.18 ± 107.90, CG = 1304.85 ± 344.07, *p* = 0.27 SUM-IPAQ- MET-min/week). No adverse effects or complications related to the exercise intervention occurred.

### Cognitive Performance Results

Mixed Linear models were applied to each test score. Results revealed that the intervention positively influenced episodic memory (PAL test) and sustained visual attention (RVP test).

Main effects of Time were detected [*F*_(__1_,_67__)_ = 5.847, *p* = 0.018] and a significant Group *x* Time interaction was detected for PAL SC [*F*_(__1_,_67__)_ = 4.038, *p* = 0.049], demonstrating that PAL was differentially affected in the CG and DTEX over time. *Post hoc* tests indicated that only the DTEx group showed significant improvements in episodic memory by increasing the number of stages completed in the test (*p* = 0.001). In contrast, there was no change for the CG (*p* = 0.790). Main effects of Time were also observed for episodic memory assessed by a decrease in Total Trials Adjusted [PAL TTA: *F*_(__1_._67__)_ = 17.079, *p* < 0.001] and an increase in the number of patterns succeeded [PAL NPS: *F*_(__1_._66__)_ = 6.647, *p* = 0.012], with significant DTEx improvements for both PAL TTA (*p* < 0.001) and PAL NPS (*p* = 0.001) while there were no changes for the CG (PAL TTA: *p* = 0.150; PAL NPS: *p* = 0.652) (Table 2).

**TABLE 2:** Cognitive performance scores on pre- and post-tests.

	Group	Pre-test	Pos-test	IC (95%)	Interaction		Time		Group	
					*F*-value	Partial $\eta_{p}^{2}$	*F*-value	Partial $\eta_{p}^{2}$	*F*-value	Partial $\eta_{p}^{2}$
Episodic memory	CG	4.07 ± 0.14	4.10 ± 0.11	−0.292 – 0.223	4.038*	0,057	5.847*	0,080	1.170	0,017
(PAL SC–score)	DTEx	4.05 ± 0.12	4.42 ± 0.10^#^***	−0.594 – −0.156						
Episodic memory	CG	5.90 ± 0.32	6.03 ± 0.25	−0.745 – 0.469	3.577	0,051	6.647**	0,091	2.506	0,037
(PAL NPS–score)	DTEx	6.03 ± 0,28	6.92 ± 0.21^##^***	−1.421 – −0.374						
Episodic memory	CG	19.83 ± 0.97	18.52 ± 0.88	−0.486 – 3.107	3.670	0,052	17.079***	0,203	0.473	0,007
(PAL TTA–score)	DTEx	20.22 ± 0.83	16.65 ± 0.75***	2.045 – 5.105						
Sustained attention	CG	713.54 ± 39.99	675.29 ± 30.63	−36.998 – 113.498	1.590	0,024	7.609**	0,103	3.476	0,050
(RVP ML–score)	DTEx	669.36 ± 36.60	566.68 ± 28.03^#^**	33.808 – 171.562						
Immediate memory	CG	16.32 ± 0.63	17.84 ± 0.70**	−2.552 – −0.480	3.246	0,044	38.534***	0,355	1.910	0,027
(Word list – CERAD)	DTEx	16.83 ± 0.55	19.58 ± 0.61***	−3.657 – −1.855						
Evocation memory	CG	4.83 ± 0.36	5.40 ± 0.38	−1.173 – 0.040	1.453	0,021	16.309***	0,191	3.778	0,052
(Word list – CERAD)	DTEx	5.46 ± 0.31	6.51 ± 0.33^#^***	−1.568 – −0.530						
Recognition memory	CG	8.64 ± 0.24	8.57 ± 0.21	−0.455 – 0.598	4.258*	0,063	2.736	0,042	6.888**	0,099
(Word list–CERAD)	DTEx	8.92 ± 0.21	9.57 ± 0.18^###^**	−1.106 – −0.191						

*Mixed linear model was used. Values are presented with mean ± SE (IC = pre-test vs. pos-test).*^#^p ≤ 0.05, ^##^p ≤ 0.01, ^###^p ≤ 0.001 (pos-test vs. pos-test); *p ≤ 0.05, **p ≤ 0.01, ***p ≤ 0.001 (pre-test vs. pos-test). *CERAD, consortium to establish a registry for Alzheimer’s disease; CG, control group; DTEx, dual-task exercise group; IC, confidence interval; PAL, paired associates learning; PAL NPS, number of patterns succeeded; PAL SC, stages completed; PAL TTA, total trials adjusted; RVP, rapid visual information processing; RVP ML, mean latency; SE, standard error*.

Taken together, these results indicate that trained participants improved cognitive performance demonstrated by the increased number of steps completed in the test (PAL SC), hit a greater number of pairs in the last step performed (PAL NPS) and decreased the required number of figures presentations (PAL TTA).

Main effects of time were also detected for sustained visual attention (RVP test) [*F*_(__1_._66__)_ = 7.609, *p* = 0.008] with no Group × Time interaction [*F*_(__1_._66__)_ = 1.590, *p* = 0.212]. *Post hoc* tests indicated significant improvements for the DTEx group as reflected by a decrease in the mean latency to correctly answer the test’s target sequence (*p* = 0.004), while there was no differences for the CG (*p* = 0.314) (Table 2).

Main effects of Group was detected for CERAD Recognition Memory [*F*_(__1_._63__)_ = 6.888, *p* = 0.01] and main effects of Time were detected on Immediate memory [*F*_(__1_._70__)_ = 38.534, *p* < 0.001] and Evocation memory [*F*_(__1_._69__)_ = 16.309, *p* < 0.001]. A significant Group × Time interaction was detected only for CERAD Recognition Memory [*F*_(__1_._63__)_ = 4.258, *p* = 0.043]. Bonferroni posttest analyses demonstrated that DTEx (*p* < 0.001) and CG (*p* = 0.005) increased the number of words on Immediate memory, however, only the DTEx group had improved performance on Evocation memory (*p* < 0.001) and Recognition (*p* = 0.006) (Table 2).

ANCOVA was applied–based on *z*-scores with adjustments by age, education, and sex–to test the hypothesis that the intervention effect sizes of DTEx could be higher than that of CG. No differences were found in Effects Size (*Z* score) between groups for Episodic memory (PAL TTA–DTEx = 0.632 ± 0.137, CG = 0.259 ± 0.161, *p* = 0.085), Sustained attention (RVP ML–DTEx = 0.464 ± 0.126, CG = 0.157 ± 0.138, *p* = 0.105), Immediate memory (DTEx = 0.831 ± 0.167, CG = 0.402 ± 0.192, *p* = 0.098), and Evocation memory (DTEx = 0.511 ± 0.159, CG = 0.314 ± 0.186, *p* = 0.424).

**FIGURE 2:**
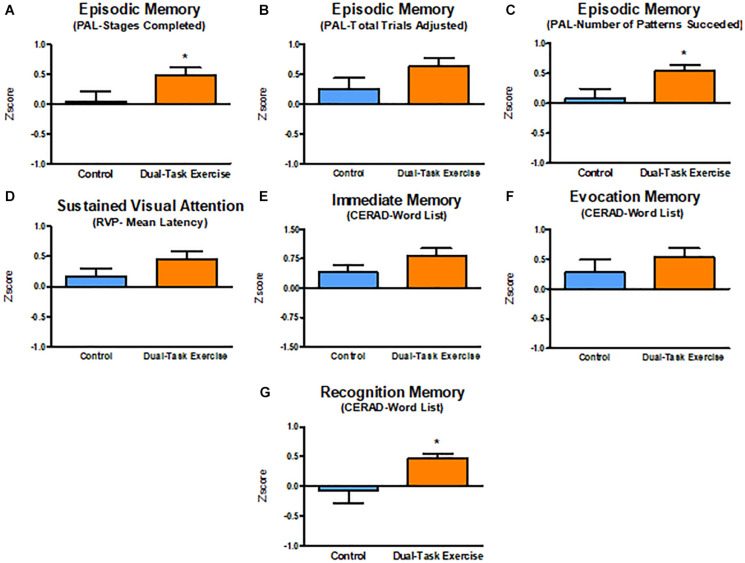
Effect sizes (*Z* score) adjusted for age, education, and sex on cognitive function. Values are presented with mean ± SE. *p ≤ 0.05 (Effect size DTEx vs. Effect size CG). CERAD, consortium to establish a registry for Alzheimer’s disease; PAL, paired associates learning; RVP, rapid visual information processing; SE, standard error. **(A–C)** Episodic Memory. **(D)** Sustained Visual Attention. **(E)** Immediate Memory. **(F)** Evocation Memory. **(G)** Recognition Memory.

DTEx group improvements showed moderate effect sizes on PAL SC (DTEx = 0.475 ± 0.126, CG = 0.064 ± 0.148, *p* = 0.039), PAL NPS (DTEx = 0.532 ± 0.114, CG = 0.093 ± 0.132, *p* = 0.015) and Recognition memory (DTEx = 0.468 ± 0.147, CG = −0.060 ± 0.169, *p* = 0.022), demonstrating significantly greater benefits of the intervention on episodic memory and recognition memory. Figure 2 illustrates effect size comparisons between groups on cognitive performance.

### Functional Exercise Capacity Results

Main effects of Time and Group, as well Group × Time interaction were detected for functional mobility [Time: *F*_(__1_._61__)_ = 7.587, *p* = 0.008; Group: *F*_(__1_._61__)_ = 13.968, *p* < 0.001; interaction: *F*_(__1_._61__)_ = 8.970, *p* = 0.004], lower limbs strength resistance [Time: *F*_(__1_._62__)_ = 42.399, *p* < 0.001; Group: *F*_(__1_._62__)_ = 25.296, *p* < 0.001; interaction: *F*_(__1_._62__)_ = 16.570, *p* < 0.001]. Significant main effects of Group and Group × Time interaction for dual-task Agility [Group: *F*_(__1_._58__)_ = 20.406, *p* < 0.001; interaction: *F*_(__1_._58__)_ = 8.315, *p* = 0.006].

Cardiorespiratory fitness improvements showed the main effects of Group [*F*_(__1_._59__)_ = 12.273, *p* = 0.001] and Group × Time interaction [*F*_(__1_._59__)_ = 14.265, *p* < 0.001]. *Post hoc* analyses indicated that the DTEx group showed significant improvements in functional exercise capacity on functional mobility (*p* < 0.001), lower limb strength resistance (*p* < 0.001), agility (*p* < 0.001), and cardiorespiratory fitness (*p* < 0.001). There were no differences from baseline for the CG (functional mobility: *p* = 0.876; lower limb strength resistance: *p* = 0.114; agility: *p* = 0.613; cardiorespiratory fitness: *p* = 0.085) (Table 3).

**TABLE 3:** Functional Exercise Capacity performance scores on pre- and post-tests.

	Group	Pre-test	Pos-test	IC (95%)	Interaction		Time		Group	
					*F*-value	Partial $\eta_{p}^{2}$	*F*-value	Partial $\eta_{p}^{2}$	*F*-value	Partial $\eta_{p}^{2}$
Cardiorespiratory	CG	406.96 ± 12.08	387.46 ± 14.27	−2.798 – 41.798	14.265***	0,195	1.105	0,018	12.273 ***	0,172
fitness (6MWT–meter)	DTEX	433.86 ± 9.73	468.40 ± 11.50^###^***	−52.499 – −16.582						
Functional mobility	CG	8.69 ± 0.30	8.73 ± 0.27	−0.528 – 0.451	8.970 **	0,128	7.587 **	0,111	13.968 ***	0,186
(TUG–second)	DTEX	7.919 ± 0.252	7.00 ± 0.224^###^***	0.508 – 1.330						
Lower limbs strength resistance	CG	9.07 ± 0.42	9.70 ± 0.40	−1.414 – 0.155	16.570***	0,211	42.399 ***	0,406	25.296 ***	0,290
(30 CST–repetition)	DTEX	10.40 ± 0.36^+^	13.13 ± 0.34^###^***	−3.400 – −2.060						
Agility	CG	1.22 ± 0.04	1.24 ± 0.04	−0.117 – 0.069	6.323 **	0,097	10.937 **	0,156	14.452***	0,197
(m/s)	DTEX	1.30 ± 0.03	1.48 ± 0.03^###^***	−0.249 – −0.099						

Mixed linear model was used. Values are presented with mean ± SE (IC = pre-test vs. pos-test). ^+^p ≤ 0.05 (pre-test vs. pre-test), ^###^p ≤ 0.001 (pos-test vs. pos-test); **p ≤ 0.01, ***p ≤ 0.001 (pre-test vs. pos-test). CG, control group; DTEx, dual-task exercise group; IC, confidence interval; SE, standard error; TUG, time up and go test; 6MWT, six-minute Walk test; 30 CST, 30-seconds chair stand test.

**FIGURE 3:**
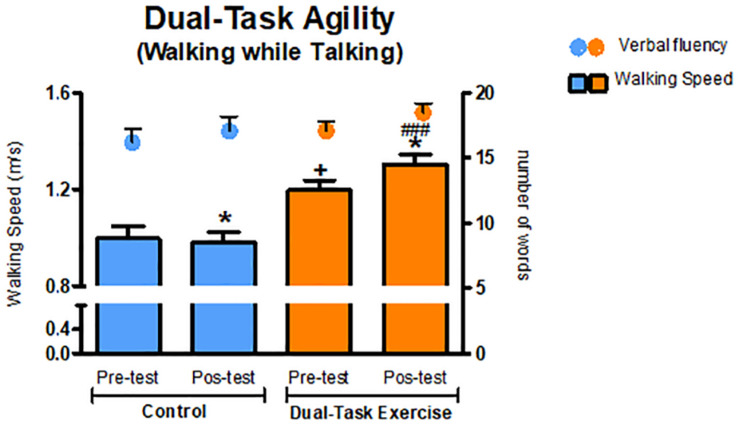
Dual task performance (*Walking while Talking Test*). Values presented as mean ± SE. ^+^*p* ≤ 0.05 (pre-test vs. pre-test); ^###^*p* ≤ 0.001 (pos-test vs. pos-test); *p ≤ 0.05 (pre-test vs. pos-test). SE, standard error.

Analysis of dual-task performance were assessed by the Walking While Talking Test. Main effects of Group [*F*_(__1_._58__)_ = 20.406, *p* < 0.001, η^2^ = 0.260] and a Group × Time interaction [*F*_(__1_._58__)_ = 8.315, *p* = 0.006, η^2^ = 0.125] were detected for dual-task agility. *Post hoc* analysis indicated significant improvements for the DTEx group on walking speed (Pre = 1.192 ± 0.043, Post = 1.320 ± 0.039, *p* = 0.039). No significant effects were detected for the cognitive component [Verbal fluency–Time: *F*_(__1_._57__)_ = 2.471, *p* = 0.122; Group: *F*_(__1_._57__)_ = 1.750, *p* = 0.191; interaction: *F*_(__1_._57__)_ = 1.115, *p* = 0.295]. These results indicate that after the intervention walking speed along with performance of a verbal fluency task was increased without declines on cognitive performance for the DTEx group. In contrast, the CG showed a reduction in dual-task agility (Pre = 1.020 ± 0.054, Post = 0.966 ± 0.050, *p* = 0.05) (Figure 3).

**FIGURE 4:**
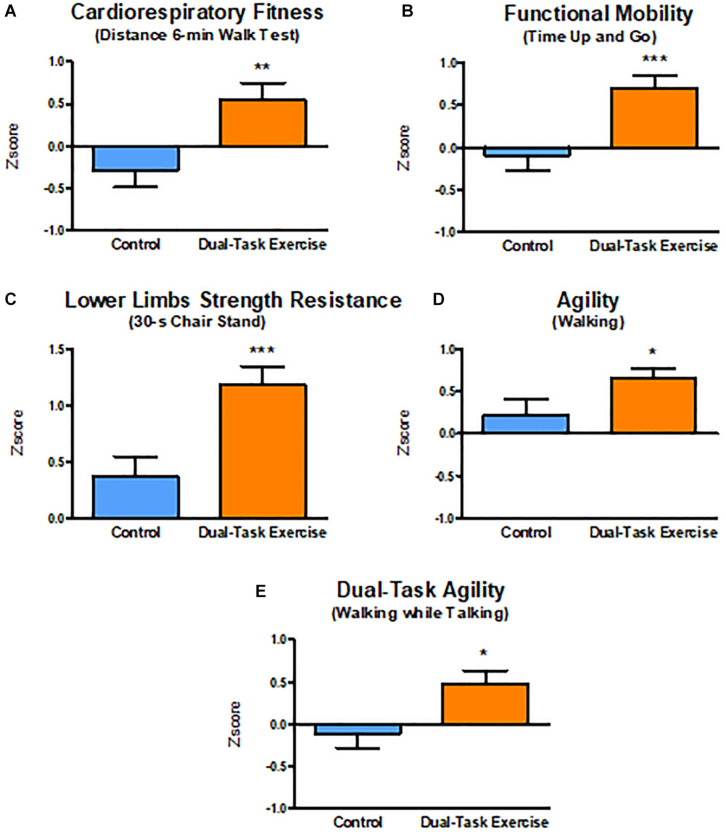
Dual Task Intervention effect sizes adjusted for age, education, and sex on Functional Exercise Capacity. Values are presented with mean ± SE. *p ≤ 0.05, **p ≤ 0.01, ***p ≤ 0.001 (Effect size DTEx vs. Effect Size CG).SE, standard error. **(A)** Cardiorespiratory Fitness. **(B)** Functional Mobility. **(C)** Lower Limbs Strength Resistance. **(D)** Agility. **(E)** Dual-Task Agility.

The DTEx group showed moderate effect sizes that were significantly greater than the CG for cardiorespiratory fitness (DTEx = 0.548 ± 0.184, CG = −0.290 ± 0.229, *p* = 0.006), functional mobility (DTEx = 0.704 ± 0.142, CG = −0.090 ± 0.170, *p* = 0.001), dual-task agility (DTEx = 0.463 ± 0.146, CG = −0.092 ± 0,185, *p* = 0.022), and agility (DTEx = 0.653 ± 0.134, GC = 0.209 ± 0.167, *p* = 0.043). The DTEx group showed a large effect size that was significantly greater than the CG for measurements of increased lower limb strength resistance (DTEx = 1.188 ± 0.148, CG = 0.368 ± 0.173, *p* = 0.001). Taken together, these results show a positive impact of the dual-task intervention program on several physical function and functional capacity measures (Figure 4).

### Quality of Life Results

Main effects of Time were detected for physical functioning [*F*_(__1_._59__)_ = 7.246, *p* = 0.009] and vitality [*F*_(__1_,_53__)_ = 5.782, *p* = 0.020] Quality of Life (QoL) domains. Effects of Time and Group [Time: *F*_(__1_._54__)_ = 11.275, *p* = 0.001; Group: *F*_(__1_._54__)_ = 5.541, *p* = 0.022] were detected on Limitations due to Physical Problems and General Health Perception domains [Time: *F*_(__1_._56__)_ = 5.273, *p* = 0.025; Group: *F*_(__1_._56__)_ = 4.050, *p* = 0.049].

The DTEx group improved in QoL related to Physical Functioning (DTEx: Pre = 75.946 ± 3.184, Post = 85.405 ± 2.251, *p* = 0.001), Role Limitations due to Physical Problems (DTEx: Pre = 70.312 ± 7.134, Post = 96.094 ± 4.985, *p* = <0.001), Vitality (DTEx: Pre = 67.000 ± 3.123, Post = 75.189 ± 3.135, *p* = 0.015), and General Health Perception (DTEx: Pre = 74.588 ± 2.981, Post = 82.441 ± 2.482, *p* = 0.018). No statistical differences were found for changes in the CG on Quality of Life assessments.

## Discussion

In this paper we analyzed older adults’ improvements on cognitive and functional capacity performances and quality of life in response to a dual-task exercise training intervention. We found there was a significant improvement in each assessment in the dual-task group after the intervention period. In contrast, the control group did not show any significant improvement in these tests. We suggest that 75 min of simultaneous multisensory cognitive stimulation and multimodal physical exercise in a dual-task paradigm, practiced regularly twice a week, may be effective at improving cognitive performance and preserving physical conditions that typically deteriorate in aging.

The hippocampus and the prefrontal cortex are affected during aging, and this may impair encoding and retrieval of episodic memory, working memory, and attention (Samson and Barnes, 2013). In contrast, regular physical exercise benefits brain structure and function, especially in aging populations, with changes occurring at molecular/cellular levels, brain structure/function, mental states, and higher order behavioral levels (Stillman et al., 2020), all of which improves functional losses (Erickson et al., 2019). Consistent with these prior results, our analysis revealed that 3 months of multimodal physical exercise training and multisensory cognitive stimulation in a dual-task paradigm improved cognitive performance on visual episodic memory, verbal episodic memory, sustained visual attention, and also improved functional capacity on cardiorespiratory conditioning, lower limb strength resistance, functional mobility, gait speed on a single task, dual-task performance and quality of life. Additionally, we estimated effect sizes (*Z* scores) to describe the proportional impact that the intervention had on cognitive and physical performance. The advantage of the *z* score as we performed is that each subject (repeated measures) has a score based on how many standard deviations the outcome has changed from baseline for each participant. These results indicated that the intervention had significantly greater moderate effects on episodic visual memory (PAL SC, PAL NPS) and verbal recognition memory (List of words) compared to the CG, while the effects of the intervention on visual attention (RVP ML), immediate verbal memory and evocation verbal memory (word list) were not different from the CG.

It is important to emphasize that few clinical trials employing dual-task training have reported effect sizes in this fashion (Desjardins-Crepeau et al., 2016; Murillo-Garcia et al., 2020). Thus, our results add to this literature an estimate for how well the intervention program worked (Cordes et al., 2019), and clinically relevant information for the efficacy of the intervention program (Brown and Ezekowitz, 2017).

It has been described that visual episodic memory and verbal episodic memory impairments are sensitive markers to identify risk for dementia (Weissberger et al., 2017) and improvements in these functions may influence daily functions necessary for autonomy (Dikmen et al., 2014; Cansino et al., 2018). Our findings demonstrate that cognitive stimulation and multimodal exercise training in a dual-task format, 75 min, twice a week, for 3 months, may have clinical importance for reducing age related cognitive decline. It has been suggested that while physical activity acts to preserve neuronal structural integrity (hardware), cognitive stimulation works on physiology and plasticity of neural circuits (software) and from these combined actions, cognitive function is amplified and prevent age-related cognitive decline and dementia (Cheng, 2016; Perneczky, 2019).

However, differently from the results shown in the present study, some studies have not found benefits using dual-task protocols (Legault et al., 2011; Ansai et al., 2017; Boa Sorte Silva et al., 2018). The absence of results may be related to the different protocol characteristics such as exercise intensity, frequency, and duration (Gheysen et al., 2018; Stojan and Voelcker-Rehage, 2019; Wollesen et al., 2020). These components seem to mediate the relationship with cognitive performance (Guo et al., 2020). Medium (12–23 weeks) or short (<12 weeks) interventions appear to be more effective in promoting cognitive improvements than longer interventions (≥24 weeks), as well as lower frequencies (≤3 session/week) compared to higher frequencies (Guo et al., 2020). However, it should be considered that some studies, even with similar duration and frequencies, present different results (Wollesen et al., 2020), which has led increasingly to consider the content of the intervention itself, such as the type of exercise and the complexity of the cognitive task.

It is also important to highlight that few studies have investigated the influence of intervention protocols on cognitive and physical domains simultaneously (Kitazawa et al., 2015; Gregory et al., 2017; Morita et al., 2018). However, considering that aging influences multiple systems and compromises the organism in a systemic way (He and Sharpless, 2017), the investigation of the physical and cognitive components are essential for the construction of programs that guarantee older adults’ autonomy (Desjardins-Crepeau et al., 2016; Tait et al., 2017; Sipilä et al., 2018; Rezola-Pardo et al., 2019). While some protocols of physical multimodal interventions including strength and balance training seem to improve components of physical performance (Kang et al., 2015), it remains inconclusive whether they also improve cognitive performance (Ansai et al., 2017; Bruderer-Hofstetter et al., 2018). More recently, the influence of dual-task training on both has been investigated, identifying benefits for cognition, but not for lower limb strength or functional mobility (Morita et al., 2018). Thus, it remains important from the point of view of construction of intervention programs using cognitive and physical stimuli in dual-tasks, to consider the benefits of the multiple dimensions of functional declines associated with aging.

In the present study, all participants had not practiced physical exercise for at least 6 months before the intervention, and during physical training had their heart rate continuously and objectively monitored, to ensure that they were meeting the recommendations of exercise intensity. We also ensured that cognitive training was sufficiently challenging and included training with episodic memory, executive function, requiring functional responses to multisensory stimuli (Lauenroth et al., 2016; Tait et al., 2017). For this, we adopted a comprehensive cognitive stimulation program previously validated (De Oliveira et al., 2014), to stimulate language, attention, decision making, episodic memory, and a variety of functional responses to multisensory stimuli.

Although we strictly followed most of the recommendations for older adult assessment (Chodzko-Zajko et al., 2009; Gregory et al., 2013; Northey et al., 2018), some cognitive skills such as sustained visual attention and immediate and delayed verbal episodic memory (Word List), did not show effect sizes significantly different from the control group, suggesting that the observed effects might not be clinically significant. In addition, we observed, as previously mentioned (Calamia et al., 2012; Scharfen et al., 2018; Adcock et al., 2020), potential practice effects, where the control group seemed to improve on measures of immediate verbal memory.

In fact, it has been shown possible practice effects resulting from the test-retest design (Jutten et al., 2020). Our results, however, demonstrated significant interaction effects for episodic memory (PAL SC) and recognition memory (Word list–CERAD) suggesting that the intervention may improve these cognitive functions. Indeed, we observed that the interaction effects were associated with different performances over time in control and dual task group. As compared with control group, the dual task group matched by age, education, and physical activity level, subjected to evaluation and revaluation in the same time window with the same cognitive tests - showed better performance. Additionally, this observation reinforces the statistical approach using effect size measurements in the present report to ensure appropriate inferences.

Important to keep in mind that learning and social interaction effects in the aging brain facing new and novel challenging tasks may also play a role in the overall cognitive and quality of life changes. This confound factor would be related to the absence of a control group designed to assess possible influences of socialization on cognitive performance and quality of life. Indeed, a recent meta-analysis based on longitudinal studies indicated that high engagement in social activity and large social networks were (weakly) associated with better late-life cognitive function (Evans et al., 2019). Thus, more randomized clinical trials are needed to investigate the potential of causal effects of socialization on cognition and clarify this confound effect (Kuiper et al., 2016).

Intervention programs using cognitive and physical exercise in dual-task paradigms seem to improve performance in functional tasks of daily life in which divided attention between tasks is required (Smith et al., 2016; Wollesen and Voelcker-Rehage, 2019). Consistent with these findings, our results revealed significant improvements when performing a test that mimics situations of daily life, in which participants should walk and speak concurrently (Dual task test “Walking While Talking”). The results showed a significant increase in the average speed used for walking in the post intervention period, whereas the control group worsened after 3 months. These results have clinical repercussions, since worse performance in dual-task situations is strongly associated with an increased risk of falls and losses of functional independence (Beauchet et al., 2009; Smith et al., 2016). Recently, this worse performance has been identified as a risk for cognitive decline and dementia (Cullen et al., 2019; Rosso et al., 2019; Åhman et al., 2019).

Gait speed is considered an important clinical parameter for geriatric evaluation (Garcia-Pinillos et al., 2016; Inzitari et al., 2017). Low gait speed is a risk factor for falls, morbidity, institutionalization, and mortality (Liu et al., 2016; Perera et al., 2016). In addition, significant increases in the usual walking speed are associated with improved survival in community dwelling, healthy older adults, with significant increases in survival with each 0.1 m/sec increase in speed (Studenski et al., 2011). The results in this study revealed that there was an increase of 13% in walking speed during a single task and 10% in a dual-task condition, suggesting that the participants increased their capacity to walk and perform a language task simultaneously.

It is worth mentioning that although the physical tests used in this study are simple, they assess habitual daily functions, which in turn are indicative of many underlying physiological processes (Jones et al., 1999; Ferguson-Stegall et al., 2017). The improvements observed in cardiorespiratory fitness, resistance of the lower limb muscle strength, functional mobility, and gait speed in dual-tasks contribute to the functional independence of older adults and are associated with quality of life (Gordon et al., 2020; Yang et al., 2020).

In previous reports (Cabral Soares et al., 2015; Bento-Torres et al., 2017; Cabeça et al., 2018) we standardized CANTAB tests procedures. Although we do not yet have a large sample to define norms and cutoff points for the Brazilian population, CANTAB tests are largely independent of cultural differences which allows appropriate comparative analysis across countries (Robbins et al., 1994; De Luca et al., 2003; Lee et al., 2013; McPhee et al., 2013). Indeed, our findings agree with a number of previous studies from other countries using CANTAB (Robbins et al., 1994, 1998; De Luca et al., 2003; Smith et al., 2013) that confirm age related cognitive decline in selected tests for both temporal and prefrontal lobe functions.

Preventive interventions dedicated to healthy older adults are important to decrease the risk of cognitive decline or dementia (Cheng, 2016). Physical exercise and/or cognitive training during life are associated with neuroprotection through an increased cognitive reserve (Stern, 2012; Perneczky, 2019). This effect may postpone or attenuate clinical manifestations in case of disease, contributing to sustained functionality and independence (Gavelin et al., 2020). In addition, it is known that high costs on dual-tasks have been associated with increased risk of developing dementia (Cullen et al., 2019; Rosso et al., 2019) and that dual-task training protocols are necessary to improve performance (Wollesen and Voelcker-Rehage, 2013). Our results showed that participants in the Dual-Task intervention group improved cognitive and physical functioning, quality of life and dual-task performance after a multimodal physical-cognitive dual-task training intervention. Our approach was designed to be reliable, effective, and feasible to be included in the primary care or seniors’ centers context, in group sessions, with limited and low-cost specific material resources.

We demonstrated that 3 months of dual-task multimodal physical exercise, 75 min/2 days per week, at moderate intensity, improves cognition, functional mobility, cardiorespiratory conditioning, upper and lower limbs strength, quality of life and dual-task performance on healthy older adults. Further investigations including intervention groups with a single stimulation, either physical training or cognitive training, for comparative purposes and blinding and randomized protocols will be necessary. In addition, longitudinal studies to investigate the detraining effects are also needed.

## Data Availability Statement

The raw data supporting the conclusions of this article will be made available by the authors, without undue reservation.

## Ethics Statement

The studies involving human participants were reviewed and approved by Health Science Institute of Federal University of Pará Review Board (CAAE no 03427318.3.0000.0018). The patients/participants provided their written informed consent to participate in this study.

## Author Contributions

CD and NB-T participated in the conception of the study, guidance, statistical analysis, data analysis, interpretation of results, and writing. JB-T contributed to the conception of the study, statistical analysis, data analysis, interpretation of results, formatting of graphics, and writing. VC participated in data collection, intervention program, data tabulation, and formatting adjustments in the text. MS contributed to writing and adjustments in the text. JC and HP contributed on data collection, tabulation, and intervention program. AT participated in data collection and tabulation. NJ participated in data collection, data tabulation, intervention program, statistical analysis, and writing. KE contributed to interpretation of results and writing. All authors have read and approved the manuscript final version and agreed with the order of presentation of the authors.

## Conflict of Interest

The authors declare that the research was conducted in the absence of any commercial or financial relationships that could be construed as a potential conflict of interest.

## Footnotes

1 http://www.gpower.hhu.de

2 https://sites.google.com/site/theipaq/scoring-protocol
